# Twenty-Second Year

**Published:** 1875-01

**Authors:** 


### TWENTY-SECOND YEAR.

### HALL'S JOURNAL OF HEALTH FOR 1875.

The Publishers desire to say that the magazine enters upon its 22d year of existence under favorable circumstances. For the last three months our circulation has averaged 12,600, and reached in December a total of 15,000 copies—a very considerable increase over the issues of the earlier months of the year. We see that our efforts to make the best and most practical health magazine, are not without appreciation, and are accordingly impelled to still further efforts to render the JOURNAL the one publication which no family can afford to be without. We strive to give an increased value to each and every number which we send out, and this effort will be in no wise remitted during the year just opening. We hope to do more this year than we have done in any single year before, in the way of imparting salutary information concerning these human houses which we inhabit—these bodies of ours—and with reference to the best methods to be used in preserving them in a sound condition. We are no enemies to the Doctors; we recognise the true glory of the healing art and its professors, but we must contribute our share towards such an education of the human race as will render physicians less a necessity than at present. Thousands of ailments which demand the doctor's attention may be avoided by temperance and right living; and these precepts we command and teach.

So we start out with the new year in the full belief that the old JOURNAL will accomplish a good deal of good before the close of 1875. To accomplish the best results, we need the assistance of all our friends. We want to enlist the aid of all who read our writings, and who appreciate our efforts to do good. We think the friendship of such of our readers as have been with us for years will prove a sufficient inducement to them to say good words in our behalf among their neighbors. There is no reader among the thousands of those who follow us from month to month, who cannot add one or more names to our list. Every act of this nature is a double service, inasmuch as it confers a lasting benefit upon the new reader thus secured, while increasing our power for good. Besides, the pleasant task of presenting the advantages of our journal to friends is not without its pecuniary reward. We have determined to deduct the sum of 50 cents from the $4, which we usually receive from two subscribers, when both are ordered by one person. Thus, twenty-five cents will be taken from each, and the two copies will be sent as desired, postage paid by us, for $3.50. If the names of three subscribers are sent, only $4.50 need accompany the order. This brings the magazine at $1.50 per year to each subscriber. Nine families of small means might club together, each contributing fifty cents, and thus secure three copies of the JOURNAL which could be passed from hand to hand until all had perused and profited by it. A good many such clubs are organized in the manufacturing districts, and we receive a great many letters from humble readers, who warmly express thanks for the practical and useful thoughts which we place before them. We have received a vast number of letters from readers who express the earnest conviction that they have been saved from prolonged illness and heavy doctors' bills by reading the JOURNAL and acting upon its suggestions.

### CLUBBING WITH OTHER PUBLICATIONS.

We call attention to the low rates at which we supply the JOURNAL in connection with other publications. If the reader will carefully examine the list, he will notice that he can secure our magazine and two others for the exact price of the two others. He will observe that a still greater saving can be made if a still larger number of periodicals are desired. We are doing a very large business in this line, and are supplying many individuals and public libraries at rates far less than they are able to make. We occupy the position of the wholesale buyer who gets the lowest possible rates by his large purchases. We supply as many as a hundred publications to a single library, and effect a very marked saving for the institution. It may appear very singular, but it is nevertheless true, that we can supply all periodicals when subscribed for in conjunction with our own JOURNAL, at a lower price than is demanded by the publishers themselves. And now a word of

